# Elevated *Plasmodium* infection rates and high pyrethroid resistance in major malaria vectors in a forested area of Cameroon highlight challenges of malaria control

**DOI:** 10.1186/s13071-018-2759-y

**Published:** 2018-03-08

**Authors:** Cyrille Ndo, Edmond Kopya, Marie Agathe Donbou, Flobert Njiokou, Parfait Awono-Ambene, Charles Wondji

**Affiliations:** 1 Malaria Research Laboratory, Organisation de Coordination pour la lutte Contre les Endémies en Afrique Centrale (OCEAC), P.O. Box 288, Yaoundé, Cameroon; 2 Vector group, Liverpool School of Tropical Medicine, Pembroke Place, Liverpool, L3 5QA UK; 3 Department of Biological Sciences, Faculty of Medicine and Pharmaceutical Sciences, University of Douala, P.O. Box 24157, Douala, Cameroon; 4 Faculty of Sciences, University of Yaoundé I, P.O. Box 337, Yaoundé, Cameroon; 5 School of Health Sciences, Catholic University of Central Africa, P.O. Box 11628, Yaoundé, Cameroon

**Keywords:** Malaria, *Plasmodium falciparum*, Pyrethroids, LLINs, *Anopheles funestus*, *Anopheles gambiae*, ELISA

## Abstract

**Background:**

High coverage of long-lasting insecticidal nets (LLINs) is the cornerstone of the malaria control strategy of the national malaria control program (NMCP) in Cameroon, with a target of reducing malaria transmission to less than 10% by 2035. To this end, more than 20 million LLINs have been distributed to populations countrywide since 2011. The present study evaluated entomological indices and *Anopheles* susceptibility to pyrethroids in a rural forested area of south Cameroon with high coverage of LLINs.

**Methods:**

The study was conducted between July 2014 and May 2016 in Obout, a village located in a rural forested area in south Cameroon. Resting mosquitoes were collected using electric aspirators and were identified to species using morphological criteria and PCR tools. Mosquito feeding preferences and infection status to *Plasmodium falciparum* were determined by ELISA and using TaqMan assays. The susceptibility of wild F1 adults to pyrethroids was monitored using WHO insecticide susceptibility bioassays.

**Results:**

During the study period, 5,993 *Anopheles* mosquitoes were collected indoors both in rooms with and without nets. Two main vector species, namely *An. funestus* and *An. gambiae*, were identified in the locality, with *An. funestus* being by far the most abundant (89.68%). ELISA analysis revealed high percentage of blood meal taken exclusively on human (97.65–98.95%) supporting the high antropohilic behaviour of both species. *Plasmodium falciparum* infection rate detected by ELISA was high throughout the study period and varied between 3.28–14.04% (mean: 10.40%) in *An. funestus*, and between 5.55–22.22% (mean: 13.87%) in *An. gambiae*. This trend was confirmed by TaqMan assays, with *P. falciparum* infection prevalence of 23.33% in *An. funestus*. Significant decrease of mortality associated with high frequency of *kdr* mutation was observed in *An. gambiae* (deltamethrin: 36.6–56.45%; permethrin: 6–18.65%) indicating high level of resistance to pyrethroids. For *An. funestus*, resistance was marked for deltamethrin (mortality: 70.54–76.24%) than for permethrin (94.12–94.74%).

**Conclusions:**

Our study showed that despite LLINs, the population of Obout remains exposed to bites of highly infected *An. funestus* and *An. gambiae* mosquitoes, highlighting the challenges to controlling malaria in forested areas, especially in the presence of insecticide resistance.

## Background

The World Health Organization set the ambitious new target of reducing the global malaria burden by 90% by 2030 [1]. Vector control is the cornerstone of that strategy through the mass distribution of free long-lasting insecticidal nets (LLINs) [2, 3]. In Cameroon, the first national campaign of massive distribution of free LLINs was conducted in 2011 and increased the proportion of households possessing at least one LLIN from 33% to 66%. However, the universal coverage, defined as one LLIN to two people at risk of malaria [4], was achieved only at 32%. Therefore, a second campaign was launched in 2015 with the aim to reach the 80% universal coverage targeted by the national malaria control program (NMCP) in the strategic plan 2011–2015 [5].

The universal coverage of LLINs has proved to be efficient in controlling malaria transmission in several sub-Saharan settings [3, 6]. However, unexpected changes have been observed in *Anopheles* vector populations in some places following mass distribution campaigns of LLINs. For example, in *An. funestus* changes in biting behaviour were observed following massive introduction of LLINs in Benin and Senegal. This vector adopted early diurnal feeding and exophagic behaviour with the proportion of mosquitoes biting outdoor increasing to 26% [7, 8]. Moreover, a change in species composition in the *An. gambiae* complex after the implementation of LLINs was noticed in Dielmo (Senegal) [9]. In *An. gambiae* populations from the same area, Trape et al. [10] reported an increase in pyrethroid resistance characterized by rise of the frequency of Leu1014Phe *kdr* resistance mutation from 8% in 2007 to 48% in 2010, after introduction of LLINs. Such changes could negatively impact malaria control operations by allowing mosquitoes to avoid contact or become resistant to insecticides.

As part of malaria control monitoring operations, it is necessary to regularly assess entomologic indices as well as level and mechanisms of insecticide resistance in vector populations in order to evaluate the effectiveness of control strategies implemented. Among the common indices recorded in vector populations are the species composition and abundance, entomological inoculation rate, blood-feeding preferences (antropophily/zoophily), biting (exophagy/endophagy) or resting (endophily/exophily) behaviour and *Plasmodium* infection rate [11, 12]. The present study aims to determine *Anopheles* species composition and abundance, anthropophily, *Plasmodium* infection rate and susceptibility profile to pyrethroids in a rural forested area of South Cameroon 3–5 years after mass distribution of LLINs.

## Methods

### Study site and period

The study started in July 2014, 3 years after the first national campaign of distribution of free LLINs, and ended in May 2016, two months after the end of the second campaign. It was conducted in Obout (3°7'N, 11°65'N), a village located in a rural forested area close to the city of Mfou, situated about 25 km from Yaoundé, the capital city of Cameroon. The vegetation around the village is constituted by an equatorial forest which is degraded by farming activities. The climate is of equatorial guinean type, characterized by two rainy seasons (August-October and April-June) and two dry seasons (November-April and June-July). The annual average rainfall is 2000 mm while average annual temperatures range between 19–29 °C, and the average humidity varies between 66–80% [13].

Obout is populated by about 200 inhabitants, most of whom are farmers. They live in houses made of mud or cement with tin rooves, presenting many interstices between the roof and the walls through which mosquitoes can enter or leave the houses. The village is also characterized by the presence of several fish ponds bordered with vegetation which could favour the development of immatures of *Anopheles* mosquito species, particularly those of *An. funestus* group. The area is known to be hyperendemic for malaria [13, 14] and the main prevention method is LLINs, with coverage of around 70% in the population.

### Mosquito collection and morphological identification

Resting mosquitoes were collected in human dwellings in the morning using electric aspirators (Rule In-Line Blowers, Model 240) and were brought back to the insectary. After species identification using morphological keys [15, 16], non-fed and some of the freshly blood engorged females were directly preserved in tubes containing desiccant for ELISA analysis to detect the presence of circumsporozoite protein (CSP) of *P. falciparum* in the head and thorax [17, 18], and to identify blood meal source [19]. The other blood-fed female mosquitoes were kept in paper cups for four days until eggs became mature. Gravid mosquitoes were allowed to oviposit according to the forced egg-laying [20], and eggs were reared to adult F1 used for insecticide bioassays, as described previously [21]. All the females were later killed and stored in tubes containing desiccant for future analysis.

### Laboratory processing of mosquitoes

Dead mosquitoes stored in Eppendorf tubes containing desiccant were divided in several parts. Wings or legs were used for genomic DNA extraction as described previously [22] and morphological identification was confirmed using PCR based assays [23, 24]. The head and thorax were used for ELISA to detect *P. falciparum* CSP [17, 18] while abdomen containing blood was used for ELISA to identify the source of blood meal [25]. Diluted *P. falciparum* sporozoite proteins supplied by the Center for Disease Control (CDC, Atlanta, USA) were used as positive controls, while ground male mosquitoes were used as negative controls. For both ELISA analyses, optical densities (OD) were read at 405 nm on an ELISA plate reader (Biotek ELx800, Swindon, UK). Positive samples were determined by OD readings 2-fold greater than the negative controls [17] and were tested a second time for validation.

Pattern of malaria transmission detected by ELISA was validated using TaqMan assay [26]. PCRs were done using the mosquito’s whole body DNA extracts and the presence of *P. falciparum* (F+) and/or *P. ovale*, *P. vivax* and *P. malariae* (OVM+) was detected in 30 field-collected *An. funestus* females. These females were randomly chosen and were different from those used for ELISA.

### Susceptibility assays to insecticides

The susceptibility of wild F1 *An. funestus* and *An. gambiae* populations to discriminating concentrations of deltamethrin and permethrin was monitored using WHO insecticide susceptibility test-kits and standard procedures [27]. Impregnated papers were obtained from a WHO reference center (Vector Control Research Unit, University Sains Malaysia, Penang, Malaysia) and their quality was first checked on the susceptible Kisumu strain of *An. gambiae*. All tests were done at a temperature of 25–27 °C and 80 ± 10% relative humidity. For each test, four batches of 20–25 unfed F1 females, 2–5 day-old, were exposed to papers impregnated with an insecticide for 1 h. Meanwhile, one batch of 20–25 mosquitoes exposed to untreated paper was used as a control. Percentage of knockdown (KD) mosquitoes was recorded at 60 min, after which mosquitoes were held for 24 h at 27 ± 2 °C and 80 ± 10% relative humidity. The kdt_50_ and kdt_95_ which correspond to the time required for knocking 50% and 95% of mosquitoes tested, were estimated using a log-time probit mode [28]. Mortalities were recorded 24 h after exposure and were compared between the two years for each species and insecticide using Fisher's exact test run in Graph Pad prims V.5. *P*-values of < 0.05 were considered as significant. Resistance/susceptible status was evaluated based on WHO criteria [27]. According to these criteria, mortality rates less than 90% were indicative of resistance while those greater than 98% were indicative of susceptibility. Mortality rates between 90–98% suggested the possibility of resistance that needs to be confirmed. Finally, 50 F0 wild *An. gambiae* mosquitoes were used for the detection of the L1014F and L1014S mutations by Taqman-kdr assay [29].

## Results

### Mosquito species composition

A total of 5993 resting *Anopheles* mosquitoes were collected during the study period (Table 1). Repartition of mosquitoes according to the presence/absence or the quality of bednet showed that 71.12% were caught in rooms without mosquito net, 26.37% in rooms with old or perforated nets, while very few (2.51%) were collected in rooms with new nets (no more than one year old). Two species groups/complexes, namely *An. gambiae* (*s.l.*) and *An. funestus* (*s.l.*), were identified according to morphological criteria, but the later was by far the most abundant representing 89.68% of the total mosquitoes caught. Molecular identification showed that *An. funestus* (*s.s.*) (hereafter *An. funestus*) and *An. leesoni* where the two species of the *funestus* group present in Obout, with *An. funestus* (*s.s.*) (98.16%, *n* = 213) being the most abundant. In the same manner, *An. gambiae* (93.96%, *n* = 109) was much more abundant compared to *An. coluzzii* (3.45%, *n* = 4); only three hybrids (2.59%) between both species were identified.

**Table 1:** Number of *An. funestus* and *An. gambiae* mosquitoes collected in Obout (Cameroon), number tested by ELISA and corresponding circumsporozoite protein rates

		2014			2015	2016			
		July	Oct.	All	Feb.	Feb.	Mar.	May	All
*An. funestus*	Collected	152	95	247	211	522	349	281	1152
	Tested	152	95	247	211	522	349	281	1152
	Positive	5	5	10	19	71	49	19	139
	ICSP (%)	3.29	5.26	4.04	9	13.6	14.04	6.76	12.07
*An. gambiae*	Collected	36	9	45	174	160	85	84	329
	Tested	36	9	45	174	160	85	84	329
	Positive	2	2	4	17	28	13	14	55
	ICSP (%)	5.56	22.22	8.89	9.77	17.5	15.29	16.67	16.72

### Blood meal source and *Plasmodium* circumsporozoite protein rate

Overall, 95.56% of mosquitoes were blood-fed, semi gravid or gravid at the time they were collected indicating that people living in surveyed houses were highly exposed to mosquito bites. ELISA analysis confirmed that *An. funestus* and *An. gambiae* were highly anthropophilic, with 97.65% (*n* = 83/85) and 98.95% (*n* = 94/95) blood meal exclusively taken on human, respectively, while less than 3% of tested samples consisted of mixed blood meals taken simultaneously on human and sheep.

Of the 2,170 head and thorax analyzed by ELISA, 245 were positive, corresponding to a high global circumsporozoite rate of 11.29%. During the study period, this rate varied between 3.28–14.04% (mean: 10.4%) in *An. funestus*, and between 5.55–22.22% (mean: 13.87%) in *An. gambiae* (Table 1). For both species, the lowest infection rates were obtained in 2014, while the highest were obtained in 2016 in mosquitoes collected after the second campaign of distribution of free LLINs.

Using TaqMan assays, 8 mosquitoes of 30 tested were found to be infected by *Plasmodium*. Among these, 7 were infected with only *P. falciparum* [F+; 23.33% (7/30)] and 1 was infected with *P. ovale*, *P. vivax* and/or *P. malariae* [OVM+; 3.33% (1/30)].

### Insecticide susceptibility

A total of 1,336 *An. funestus*, 822 *An. gambiae* and 546 females from the *An. gambiae* Kisumu strain were used in WHO susceptibility tests using two pyrethroids (permethrin and deltamethrin) (Table 2).

**Table 2:** Mortality and knockdown time of *An. funestus* and *An. gambiae* from Obout (Cameroon) after 1 h exposure to pyrethroids

Year	Species/strain	Insecticide	*n*	kdt_50_ (min) (IC_95_)	kdt_95_ (min) (IC_95_)	Mortality (%)	Status
2015	Kisumu	Perm. 0.75%	120	21.2 (16.1–25)	40 (33.88–55.5)	100	S
		Delta. 0.05%	100	18.1 (17.6–18.9)	24.9 (23.4–27.4)	100	S
	*An. funestus*	Perm. 0.75%	152	17.2 (16.8–17.7)	22.4 (21.4–23.8)	94.74^a^	PR
		Delta. 0.05%	101	26.6 (18.2–39.9)	55.2 (37.7–214.7)	76.24^b^	R
	*An. gambiae*	Perm. 0.75%	153	> 60	>> 60	36.6^c^	R
		Delta. 0.05%	124	38.3 (35–41)	>> 60	56.45^e^	R
2016	Kisumu	Perm. 0.75%	167	21.12 (16.1–24.6)	40.3 (33.88–55.5)	100	S
		Delta. 0.05%	159	18 (17.6–18.9)	24.9 (23.4–27.4)	100	S
	*An. funestus*	Perm. 0.75%	510	29.41 (22.52–38.4)	41.61 (32.15–52.86)	94.12^a^	PR
		Delta. 0.05%	573	39.62 (20.6–54.19)	55.19 (44.89–90.19)	70.54^b^	R
	*An. gambiae*	Perm. 0.75%	202	> 60	>> 60	6.00^d^	R
		Delta. 0.05%	343	> 60	>> 60	18.65^f^	R

^a-f^ For *An. funestus* and *An. gambiae* mortality rates for the same insecticide followed by different letters were significantly different between the two years (Fisher's exact test). All significant differences were at *P* < 0.0001.

*Abbreviations*: kdt, Knockdown time; R, resistant; S, susceptible; PR, probably resistant; Perm., permethrin; Delta., deltamethrin.

**Fig. 1:**
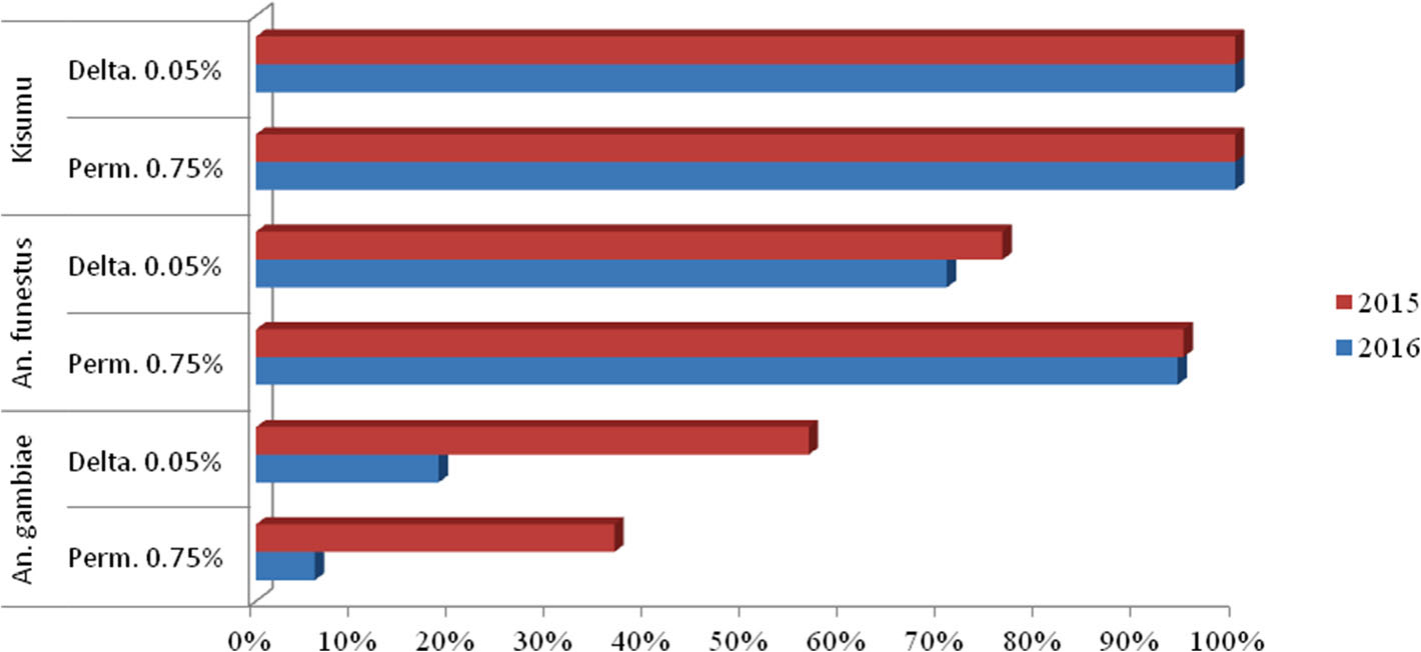
Trends of mortality to permethrin and deltamethrin in *An. funestus* and *An. gambiae* mosquitoes from Obout (Cameroon) in 2015 and 2016

The *An. gambiae* Kisumu strain displayed fully susceptible phenotype for all insecticides tested with KDT_50_ less than 30 min and 100% mortality, indicating that the impregnated papers were of good quality. By contrast, high level of resistance to permethrin and deltamethrin, characterized by significant decrease of mortality coupled with increase in knockdown time, was observed in *An. gambiae*. This resistance significantly increased one year to another [(Fisher's exact test for permethrin: *P* < 0.0001; OR: 9.141; CI: 4.68–17.86 ); Fisher's exact test for deltamethrin: *P* < 0.0001; OR: 5.124; CI: 3.82–8.00)] with mortality of 6% and 18.65% to permethrin and deltamethrin, respectively, in 2016 compared to 36.6% and 56.45%, respectively, in 2015. (Table 2, Fig. 1). Using TaqMan-kdr assay, both L1014F and L1014S *kdr* mutations were identified in *An. gambiae* population from Obout. However, the frequency of the L1014F (98.72%) mutation was very high compared to that of the L1014S (7.95%) mutation.

In *An. funestus*, the level of resistance to deltamethrin was moderate, but also increased one year to another, with mortality rates of 76.24% and 70.54% in 2015 and 2016, respectively, but the difference was not significant [(Fisher’s exact test: *P* = 0.283; OR: 1.342; CI: 0.82–2.19)]. For permethrin, low mortality rates (94.12–94.74%), suggesting probable resistance were recorded throughout the study period. Contrary to *An. gambiae*, the resistance to pyrethroid in *An. funestus* was not associated with elevation of KDT_50_, suggesting that *kdr* is probably not involved in the resistance to pyrethroids in this species (Table 2, Fig. 1).

## Discussion

The present study highlights important malaria transmission due to *An. funestus* and *An. gambiae* in a rural forested area of South Cameroon. Both species were present in the village throughout the year and were highly infected by *P. falciparum*, with infection rates reaching 22%. However, the densities of *An. funestus* populations were significantly higher over the study period, making this species the major malaria vector in this locality, where it probably breeds in artificial fish ponds situated around the village.

*Anopheles* infection status was mainly determined by detecting the presence of *P. falciparum* circumsporozoite protein in the head and thorax of mosquitoes by ELISA. Although this technique could overestimate *Plasmodium* sporozoite prevalence by a factor of 1.1–1.9 in mosquitoes [30], the fact that TaqMan assays also detected high *P. falciparum* infection prevalence in *An. funestus* (2-fold than ELISA) reinforces the view that level of malaria transmission in the surveyed locality remains high, despite high coverage of LLINs.

LLIN represents the tool of choice for malaria control. It has significantly contributed to substantial reduction of malaria transmission in sub-Saharan African countries since its vulgarisation in 2000 [3, 6]. LLIN plays a double role by protecting humans from mosquito bites and by killing mosquitoes which come in contact with the net. In this study, new mosquito nets received during the 2015–2016 national campaign of distribution of free LLINs were effective at preventing mosquito bites, since very few and non-fed mosquitoes were collected in rooms with new nets. In contrast, the fact that a non-negligible proportion (26.37%) of blood-fed mosquitoes were collected in rooms with old LLINs received during the first national campaign of distribution of free LLINs in 2011 indicated that they could have lost their efficacy, probably due to several inappropriate washing or net deterioration. This highlights the need to replace LLINs that are torn or show waning efficacy to sustain high level coverage, in order to effectively reduce malaria transmission in sub-Saharan Africa [4, 30]. Regarding this, the WHO recommends that malaria endemic countries should supply LLINs through a combination of mass free distribution campaigns, normally at interval of no more than three years, and continuous distributions particularly during immunisation and antenatal services [31].

In addition, the high level of resistance to pyrethroids observed in malaria vectors in this study could have also contributed to the loss of net efficacy by allowing resistant mosquitoes to enter the nets or to bite through holes. Although our results showed that the *kdr* L1014F mutation, and to lesser extend the L1014S mutation, were involved in the resistance observed in *An. gambiae*, it could not be biased to assume *kdr* allele alone conferred the ability to survive diagnostic doses of pyrethroids. Thus, metabolic mechanisms [32, 33] must also contribute to the high-level of pyrethroid resistance in this species. This would also be the case for *An. funestus*, which exhibited moderate and probable resistance to delthamethrine and permethrin, respectively, owing to the fact that *kdr* was never reported in this species. Similar to this study, difference in levels of insecticide resistance between sympatric populations of the two vector species was already reported elsewhere [34] and could be explained by difference in their biological characteristics. *Anopeheles gambiae* breeds in temporary stagnant waters, which are more polluted by agricultural pesticides, but less for *An. funestus* for which larvae develop in large semi-permanent water. Another main difference among the species could come from the fact that *An. funestus* probably recently colonized the surveyed locality, as fish ponds were less than three years old, and thus has not yet receive enough insecticide pressure selection. Further investigations are necessary to fully elucidate key insecticide resistance mechanisms for *An. gambiae* and *An. funestus* in our study area. Nevertheless, such a high level of resistance to the two pyrethroids used for net impregnation for major malaria vectors is a concern for the continued effectiveness of this key malaria control tool, and this call for an urgent development of new insecticide compounds with different mode of action [35].

Beside issues of insecticide resistance, partial coverage LLINs could also seriously limit the efficacy of malaria control operations in highly endemic settings such as forested areas of Cameroon. In fact, non-covered areas or houses without nets could represent hot spots of malaria transmission while people living in these places could serve as reservoir of *Plasmodium* parasites [36]. This hypothesis could also explain why a drop in malaria transmission (*Anopheles* infection) was not observed in the present study, even after the second campaign of distribution of LLINs. Our analysis therefore points out the necessity to increase LLINs coverage across Cameroon. and other control measures should be combined to LLINs in order to achieve the goal of reducing malaria cases to less than 10% by 2030. These include indoor residual spraying (IRS), symptomatic diagnosis and treatment of malaria cases using artemisinin-based combination therapy (ACT) especially for children under 5 years, and prevention and control of malaria during pregnancy by administration of intermittent preventive treatment (IPTp) using Sulfadoxine-Pyrimethamine (SP). Moreover, since fish ponds represented major larval breeding sites in our studied area, perhaps combining larval control with LLINs should be considered.

## Conclusions

The results of this study showed that the population of Obout sleeping in rooms without net or rooms with only old nets were highly exposed to bites of highly infected and pyrethroid resistant *An. funestus* and *An. gambiae* mosquitoes. In the context where malaria elimination is back again on the agenda of WHO and various stakeholders, the present study highlights the importance of achieving universal coverage of LLINs, the need to replace used LLINs two to three years after their distribution, and the necessity to implement additional malaria control measures in our study site. Meanwhile, more attention must be paid on the evolution of insecticide resistance in *Anopheles* vector species, which could seriously impede malaria control operations based on the use of insecticide or insecticide-treated tools including LLINs. Further studies are also necessary in order to investigate all factors which could explain such high level of malaria transmission despite large coverage of LLINs, by assessing human behaviour and use of LLINs, resting and biting behaviour of malaria vectors in the locality as well as mechanisms involved in insecticide resistance.

## Abbreviations

CSP: circumspororzoite protein
DDT: dichlorodiphenyltrichloroethane
ELISA: enzyme-linked-immunosorbent assay
IPTp: intermittent preventive treatment
IRS: indoor residual spraying
KD: knockdown
KDT: knockdown time
LLINs: long-lasting insecticidal nets
OD: optical density
PR: probably resistant
R: resistant
S: susceptible
SP: sulfadoxine-pyrimethamine
WHO: World Health Organization
